# Impact of obstructive lung disease and sleep apnea symptoms on cardiovascular risk and all-cause mortality: insights from a community-dwelling cohort

**DOI:** 10.1007/s11325-025-03564-0

**Published:** 2026-01-05

**Authors:** Trygve M. Jonassen, Bjørn Bjorvatn, Sahrai Saeed, Tomas M. Eagan, Sverre Lehmann

**Affiliations:** 1 Centre for Sleep Medicine, Department of Thoracic Medicine, Haukeland University Hospital, Postboks 1400, Bergen, 5021 Norway; 2 Department of Global Public Health and Primary Care, University of Bergen, Bergen, Norway; 3 Norwegian Competence Center for Sleep Disorders, Haukeland University Hospital, Bergen, Norway; 4 Department of Cardiology, University of Oslo, Oslo, Norway; 5 Department of Clinical Science, University of Bergen, Bergen, Norway

**Keywords:** Obstructive sleep apnea, Chronic obstructive pulmonary disease, Overlap syndrome, Cardiovascular risk, Mortality

## Abstract

**Purpose:**

Chronic obstructive pulmonary disease (COPD) and obstructive sleep apnea (OSA) are prevalent conditions with overlapping pathophysiological mechanisms. Their coexistence, termed overlap syndrome, is thought to amplify cardiometabolic risk. This study examined the 10-year risk of major adverse cardiovascular events (MACE) and all-cause mortality in individuals with COPD and OSA symptoms in a community-based cohort.

**Methods:**

Baseline data (1998–1999) from the Hordaland Health Study were linked to national registries on mortality and cardiovascular events. Of 7,456 eligible adults born 1925–1927 and 1950–1951, a random sample of 5,100 was invited, and 3,305 with valid spirometry were included. OSA symptoms were assessed by questionnaire, and chronic airway obstruction (CAO) was defined as post-bronchodilator FEV₁/FVC < 0.70. Cox regression estimated hazard ratios (HR) for MACE and all-cause mortality.

**Results:**

CAO independently predicted both MACE (HR 1.48, 95% CI 1.12–1.97, *p* < 0.006) and all-cause mortality (HR 1.78, 95% CI 1.44–2.22, *p* < 0.001). Excessive daytime sleepiness (EDS) was associated with increased mortality (HR 1.37, 95% CI 1.01–1.85, *p* = 0.045). A significant interaction was found between CAO and habitual snoring, with participants displaying both having more than a twofold increased risk of mortality (HR 2.22, 95% CI 1.31–3.76, *p* = 0.003).

**Conclusions:**

CAO and EDS emerged as independent predictors of mortality, while the coexistence of CAO and snoring conferred synergistic risk. These findings highlight the need to recognize OSA symptoms in patients with obstructive lung disease, as they may identify a vulnerable subgroup at heightened risk. Future studies using objective sleep assessments are warranted to clarify mechanisms and guide preventive strategies.

## Introduction

Chronic obstructive pulmonary disease (COPD) is a global cause of significant morbidity and mortality, accounting for approximately three million deaths annually [1]. Using the spirometric criterion of a post-bronchodilator FEV_1_/FVC ratio below 0.7 to diagnose COPD [2], an estimated 392 million individuals aged 30 to 79 had COPD in 2019, representing a global prevalence of 10.3% [3]. Obstructive sleep apnea (OSA), a disorder characterized by “narrowing of the upper airway that impairs normal ventilation during sleep” [4], is an even more prevalent condition, with 425 million people aged 30–69 having moderate and severe OSA [5]. Severe OSA has been linked to increased cardiovascular and all-cause mortality in several epidemiological studies including more than 24000 subjects [6]. Like OSA, COPD is associated with cardiac comorbidities, and mortality in moderate COPD is more frequently due to cardiovascular events than respiratory complications [7].

The overlap of COPD and OSA, known as the overlap syndrome, is characterized by combined pathophysiological features, including periodic or constant hypoxemia, hypercapnia, systemic inflammation, oxidative stress, endothelial dysfunction, and sympathetic activation [8–10]. These mechanisms are believed to exert synergistic effects, accelerating the development of pulmonary hypertension, heart failure, and atherosclerosis [10]. The occurrence of this syndrome is estimated to affect 1–2% of adults in the general population but may be much higher in clinical settings [11]. Given the shared pathophysiological mechanisms of COPD and OSA, the overlap syndrome should be expected to confer an increased risk of cardiovascular disease and mortality [10]. Indeed, several studies have reported that individuals with overlap syndrome are more likely to develop pulmonary hypertension [12], heart failure [13], hypertension [14], and COPD exacerbations and death [15], compared with those with COPD alone. Notably, although the referenced study on COPD exacerbations and death was not randomized, it suggested that patients with overlap syndrome, when effectively managed with continuous positive airway pressure (CPAP), exhibit risks like those with COPD alone in terms of the clinical outcomes assessed [15]. However, other investigations have produced conflicting findings, failing to demonstrate that overlap syndrome carries greater cardiovascular risk than either disorder in isolation [16, 17].

Population-based studies on the risk of cardiovascular events and mortality in coexistent COPD and OSA are few. In the 11-year follow-up community-based Sleep Heart Health Study (SHHS) of 6173 middle-aged and older U.S. adults, home sleep testing was applied for the diagnosis of OSA. Contrary to the study hypothesis, interaction analyses revealed that the attributable risk of a low forced expiratory volume in one second (FEV_1_) to all-cause mortality decreased with increasing OSA severity [17]. In a study examining data from the National Health and Nutrition Examination Survey (year 2005–2008), which employed questionnaire-based diagnoses for both COPD and OSA, individuals with OSA-COPD overlap syndrome had a higher risk of all-cause mortality, compared to those with COPD alone. However, this risk increase did not achieve statistical significance after a 7-year follow-up among 10,388 participants aged 20 years or older [18].

The aim of the current study was to examine the 10-year risk of cardiovascular events and all-cause mortality by COPD, defined by chronic airway obstruction (CAO), and self-reported symptoms of OSA in middle-aged and older adults from the general population. We hypothesized that CAO and OSA symptoms would independently predict adverse outcomes, and that their coexistence would confer synergistic risk.

## Study design and methods

### Study design

**Fig. 1:**
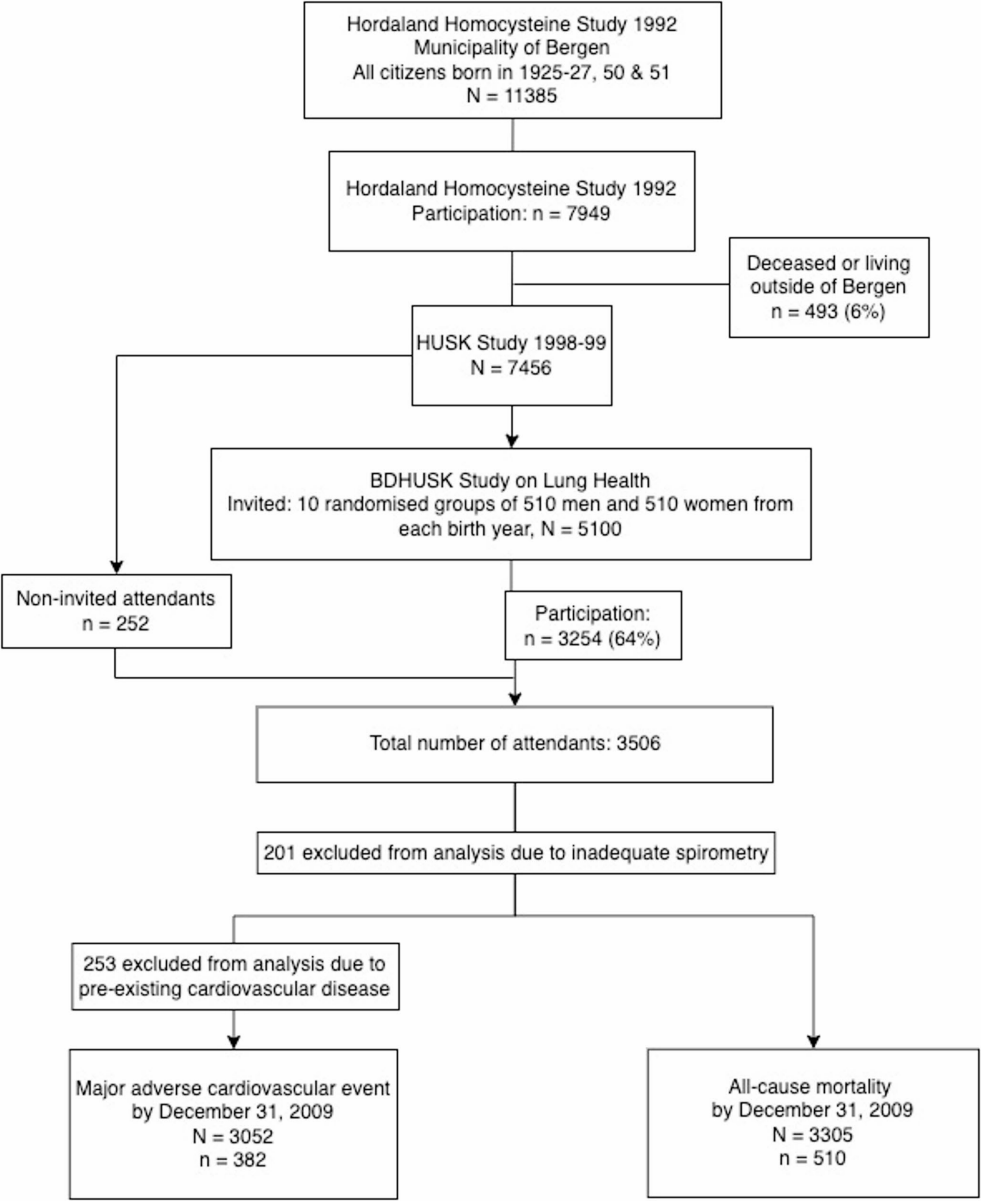
Flow diagram of participant recruitment, exclusions, and analytic samples. Of 7,949 individuals born in 1925–1927 and 1950–1951 who participated in the earlier Hordaland Health Study (1992–1993), 493 were deceased or had relocated, leaving 7,456 eligible. A random sample of 5,100 was invited, of whom 3,506 attended baseline examinations in 1998–1999. After excluding 201 participants with inadequate spirometry, **3**,**305 individuals were included in the all-cause mortality analyses**. For the major adverse cardiovascular event (MACE) endpoint, an additional 253 participants with pre-existing cardiovascular disease at baseline were excluded, leaving **3**,**052 individuals eligible for analysis**

Cross-sectional data from the population-based Hordaland Health Study (HUSK) collected in 1998–1999 were merged with prospective data from the Norwegian Cause of Death Registry (CoDR), and the Cardiovascular Disease in Norway project (CVDNOR). The regional ethics committee of Western Norway approved the study (REK-Vest number 2010/2560). A flow diagram of participant enrollment and inclusion in endpoint analyses is provided in Fig. 1.

### Population

The study group included all individuals born in the years 1925 to 1927 and 1950 to 1951, residing in Bergen, Norway, who had participated in an earlier phase of the HUSK study during 1992–1993 (*n* = 7949, participation rate: 67%) [19]. Of the 7949 individuals eligible for the study, 493 had either passed away or relocated from the area. Among the remaining 7456 individuals, random selections of 510 women and 510 men from each birth year (*n* = 5100) received an invitation via postal mail. Ultimately, 3506 participants (1780 women and 1726 men, 69% of invited subjects) responded and underwent examination at the HUSK study center.

### Baseline data

OSA symptoms were assessed utilizing a subset of three questions from the Karolinska Sleep Questionnaire [20]. These self-reported inquiries pertained to frequency of snoring, instances of breathing pauses during sleep, and excessive daytime sleepiness (EDS). The optional answers to these questions were “never”, “seldom”, “sometimes”, “often”, and “always”. Participants who answered either “often” or “always” were considered positive for the single symptom in question, whereas participants who responded either “sometimes”, “often”, or “always” on all three items were classified as “OSA cases”. A comparable definition, based on the Hawaii Sleep Questionnaire (the apnea score), has previously undergone validation against polysomnography. This validation revealed its ability to correctly identify 100% of cases with severe OSA (defined as an apnea-hypopnea index (AHI) of ≥ 40). For an AHI threshold of ≥ 10, the questionnaire demonstrated a sensitivity of 78% and a specificity of 67% [21].

Data on smoking habits, use of antihypertensive medication, diabetes, and any previous cardiovascular disease were also collected using questionnaires.

Spirometry, which included a bronchodilator test involving the inhalation of 400 µg of Salbutamol, was conducted for all participants. All tests were administered by a single, highly trained technician. A total of 201 subjects were excluded from the analysis due to inadequate spirometry performance, as per ATS 1994 criteria. This exclusion left 3305 cases eligible for all subsequent analyses. Details regarding the characteristics of the excluded subjects (*n* = 201) have been previously documented [22]. In all conducted analyses, the results for post-bronchodilator FEV_1_, forced vital capacity (FVC), and the FEV_1_/FVC ratio were utilized. Predicted values for FEV_1_ and FVC were calculated by applying the 2012 reference equations from the Global Lung Function Initiative (GLI). These calculations were done using the European Respiratory Society’s GLI calculator [23]. Subjects with a post-bronchodilator FEV_1_/FVC ratio < 0.70 were considered as having CAO. This fixed threshold was selected as it represents the diagnostic criterion for COPD recommended by the Global Initiative for Chronic Obstructive Lung Disease (GOLD) both at the time of baseline data collection [24] and in the current GOLD 2023 report [2]. The use of post-bronchodilator values is essential to distinguish between asthma-related reversible obstruction and chronic, irreversible obstruction characteristic of COPD. Applying this definition ensures consistency with international standards and comparability with other large epidemiological studies of COPD. In this study, we used CAO as a spirometric proxy for COPD, recognizing that while not identical, this operational definition is consistent with GOLD diagnostic criteria and widely applied in epidemiological research [25].

Measurements of height, weight, waist circumference, and hip circumference were taken for all participants. From these measurements, body mass index (BMI) and waist-hip ratio were computed. Obesity was defined as BMI ≥ 30 kg/m^2^, or waist-hip ratio ≥ 0.85 in women and ≥ 0.90 in men, as recommended by the World Health Organization [26].

Baseline measurements also included clinic blood pressure (BP), and non-fasting analyses of serum total cholesterol and high-density lipoprotein (HDL) cholesterol. Hypertension was defined as either systolic BP of ≥ 140 mmHg and/or diastolic BP of ≥ 90 mmHg, based upon clinic BP values. A total cholesterol level of 7.0 mmol/l or more was defined as elevated. For HDL cholesterol, levels below 1.3 mmol/l and 1.0 mmol/l were considered low in women and men, respectively.

### Outcomes

The two primary endpoints were occurrence of a major adverse cardiovascular event (MACE), and all-cause mortality. MACE was defined as a composite of nonfatal myocardial infarction (MI), nonfatal stroke, and cardiovascular death. CVDNOR contains data on all cardiovascular disease related hospital admissions in Norway from 1994 until the end of 2009, and from this dataset the first occurrence of a nonfatal MI or a nonfatal stroke was identified. Individuals who at baseline had reported a history of previous MI or stroke, or who had had a hospital admission with MI or stroke prior to baseline, were excluded from MACE analysis. Cases of cardiovascular deaths were identified by death certificate diagnoses, recorded in CoDR. In addition, CoDR was the sole source of data for all-cause mortality events. Date of censoring for both endpoints was December 31, 2009.

### Diagnostic definitions and thresholds

All categorical variables and diagnostic criteria used in this study were defined as detailed below:

- Chronic airflow obstruction (CAO): post-bronchodilator FEV₁/FVC < 0.70, measured 20 min after inhalation of 400 µg salbutamol, in accordance with GOLD recommendations.
- Obesity: body mass index (BMI) ≥ 30 kg/m².
- Central obesity: waist–hip ratio ≥ 0.85 in women and ≥ 0.90 in men.
- Hypertension: systolic blood pressure ≥ 140 mmHg and/or diastolic blood pressure ≥ 90 mmHg.
- Low HDL cholesterol: < 1.3 mmol/L in women and < 1.0 mmol/L in men.
- Elevated total cholesterol: ≥ 7.0 mmol/L.
- Diabetes: self-reported physician diagnosis.
- Smoking status: categorized as never, former, or current smoker.
- OSA symptoms: snoring, witnessed breathing pauses, and excessive daytime sleepiness were defined as present when participants reported the symptom “often” or “always.”
- “OSA cases”: defined as reporting all three symptoms (“sometimes”, “often” or “always”).
- Exclusion criteria: participants with inadequate spirometry were excluded from all endpoint analyses; those with prior cardiovascular disease at baseline were excluded from MACE analyses.

### Statistical analyses

 For both outcome variables (MACE and all-cause mortality), initial analyses examined various categorical explanatory factors. Significance was tested with two-sided chi-square tests. Hazard ratios (HR), both crude and after adjusting for covariates, were estimated using Cox regression analyses. These covariates included age, sex, waist-hip ratio, smoking history, hypertension, diabetes, total cholesterol, HDL cholesterol, and CAO (FEV_1_/FVC < 0.7), and they were accounted for in all multivariate analyses. The three symptoms of OSA, and “OSA cases”, were entered separately to the regression equations. Furthermore, to delve deeper into whether the coexistence of obstructive lung disease and symptoms of OSA was associated with an elevated risk of cardiovascular disease or overall mortality, interaction analyses were conducted for both endpoints. These analyses included all potential interactions between CAO and the various symptoms of OSA. All statistical analyses were carried out using SPSS version 26, with a predetermined level of statistical significance set at 5%. To reduce the likelihood of Type I error due to multiple testing, the threshold for statistical significance in the interaction analyses was adjusted to 1%.

## Results

In total, 3305 participants were included in the analysis, of which 49.5% (1635) were men. Approximately 60% of the study population were either current (*n* = 788) or former smokers (*n* = 1248), 9.2% had CAO defined by a post-bronchodilator FEV_1_/FVC ratio below 0.7, and 5.4% had a history of cardiovascular disease. Among the self-reported symptoms of OSA, snoring was the most prevalent at 17.0%, followed by daytime sleepiness and breathing cessations with respective prevalences of 10.4% and 3.0%. In total, 4.8% of the study sample were classified as “OSA cases”.

Table 1 details baseline characteristics of the population, categorized by age group and sex. In the middle-aged cohort, men exhibited a significantly higher prevalence of obesity, defined by an elevated waist-hip ratio, but no significant sex difference was observed when obesity was assessed using BMI. Hypertension was also significantly more prevalent in men. In the middle-aged cohort, no significant distinctions between sexes were noted in terms of diabetes prevalence, cholesterol levels, smoking habits, or spirometry results. However, significant differences emerged for all self-reported symptoms of OSA, with snoring and breathing cessations being more common in men, and daytime sleepiness more prevalent in women (Table 1).

**Table 1:** Baseline characteristics of the study population within age and sex strata, *N* = 3305

	Middle-aged (47–48 yrs.),n = 1505			Older (71–73 yrs.),n = 1800		
	Women	Men		Women	Men	
	n (%)	n (%)	p*	n (%)	n (%)	p*
*Obesity*						
Body mass index ≥ 30 kg/m^2^	81 (10.1)	70 (9.9)	0.89	142 (16.3)	87 (9.4)	< 0.01
Waist-hip ratio^a^	173 (21.7)	422 (59.9)	< 0.01	325 (37.4)	759 (81.7)	< 0.01
*Smoking history*						
Never	288 (36.8)	242 (34.7)	0.32	488 (57.5)	190 (20.7)	< 0.01
Ex	217 (27.7)	218 (31.3		232 (27.4)	581 (63.4)	
Current	278 (35.5)	237 (34.0)		128 (15.1)	145 (15.8)	
*Hypertension* ^b^						
Normotensive, no BP medication	666 (83.4)	503 (71.3	< 0.01	304 (34.9)	295 (31.8)	0.37
Normotensive, on BP medication	21 (2.6)	15 (2.1)		48 (5.5)	64 (6.9)	
Hypertensive, on BP medication	15 (1.9)	14 (2.0)		182 (20.9)	192 (20.7)	
Hypertensive, no BP medication	97 (12.1)	174 (24.6)		337 (38.7)	378 (40.7)	
*Diabetes*						
No	795 (99.5)	696 (98.6)	0.10	826 (94.8)	862 (92.8)	0.07
Yes	4 (0.5)	10 (1.4)		45 (5.2)	67 (7.2)	
*Serum cholesterol*						
Total cholesterol ≥ 7 mmol/l	67 (8.4)	79 (11.2)	0.07	310 (35.7)	132 (14.2)	< 0.01
Low HDL cholesterol^c^	252 (31.6)	205 (29.0)	0.28	295 (33.9)	232 (25.0)	< 0.01
*Spirometry (post-bronchodilator)* ^d^						
FVC ≥ 80%	736 (92.1)	646 (91.5)	0.31	739 (84.8)	766 (82.5)	0.02
50% ≤ FVC < 80%	63 (7.9)	58 (8.2)		129 (14.8)	159 (17.1)	
FVC < 50%	0	2 (0.3)		3 (0.3)	4 (0.4)	
FEV_1_ ≥ 80%	746 (93.4)	645 (91.4)	0.23	740 (85.0)	728 (78.4)	< 0.01
50% ≤ FEV_1_ < 80%	52 (6.5)	58 (8.2)		119 (13.7)	175 (18.8)	
FEV_1_ < 50%	1 (0.1)	3 (0.4)		12 (1.4)	26 (2.8)	
FEV_1_/FVC ≥ 0.7	783 (98.0)	682 (96.6)	0.09	798 (91.6)	739 (79.5)	< 0.01
FEV_1_/FVC < 0.7	16 (2.0)	24 (3.4)		73 (8.4)	190 (20.5)	
*Symptoms of sleep apnea*						
Snoring	97 (12.1)	184 (26.1)	< 0.01	98 (11.3)	183 (19.7)	< 0.01
Breathing cessations	13 (1.6)	34 (4.8)	< 0.01	18 (2.1)	33 (3.6)	0.06
Daytime sleepiness	131 (16.4)	83 (11.8)	0.01	68 (7.8)	61 (6.6)	0.31
“OSA cases”^e^	28 (3.5)	65 (9.2)	< 0.01	17 (2.0)	47 (5.1)	< 0.01
*Pre-existing cardiovascular disease*						
Stroke	4 (0.5)	3 (0.4)	0.83	24 (2.8)	48 (5.2)	< 0.01
Myocardial infarction	0	12 (1.7)	< 0.01	54 (6.2)	122 (13.1)	< 0.01

*Chi-Square test, except for variables with cell counts below 5 (Fischer’s exact test). BP, blood pressure; HDL, high-density lipoprotein; FVC, forced vital capacity; FEV1, forced expiratory volume in 1 second; OSA, obstructive sleep apnea

^a^Cut-offs of 0.85 and 0.90 were used to define obesity in women and men, respectively

^b^Hypertension defined by measured systolic blood pressure of ≥140 mmHg and/or diastolic blood pressure of ≥90 mmHg

^c^Low HDL cholesterol was defined by levels below 1.3 mmol/l in women, and by levels below 1.0 mmol/l in men

^d^Spirometry was done 20 minutes after inhalation of 400 µg Salbutamol

^e^"OSA cases" defined by self-reported co-occurrence of snoring, breathing cessations, and daytime sleepiness

In the older age group, significant differences between women and men were found for all variables except hypertension, diabetes, FVC, breathing cessations and daytime sleepiness. Smoking, elevated total cholesterol, low HDL cholesterol, obstructive lung function impairment, snoring, and a history of stroke or myocardial infarction, were all more prevalent in men than in women. Notably, conflicting results were observed in obesity measurements depending on the applied definition. Specifically, obesity, defined by a BMI of 30 or higher, was nearly twice as prevalent in women compared to men. However, an elevated waist-hip ratio was significantly more common in men than in women (Table 1).

During a mean follow-up period of 9.9 years for MACE, a total of 382 events were recorded. Meanwhile, for all-cause mortality, the follow-up averaged 10.3 years, during which 510 events were observed.

Bivariate analyses between the different explanatory variables and the 10-year incidence of MACE and all-cause mortality are presented in Table 2. Increasing age, male sex, former or current smoking, hypertension, diabetes, and CAO all correlated significantly with both the occurrence of MACE and with all-cause mortality. Obesity correlated strongly with both endpoints, when defined by the waist-hip ratio, but for BMI no statistically significant relationship was found. Elevated total cholesterol was a risk factor for MACE but was not significantly associated with all-cause mortality. Low HDL cholesterol was not significantly associated with either endpoint. There were no statistically significant associations between any of the OSA symptoms and all-cause mortality, but negative correlations were found between two of the symptoms (breathing cessations, daytime sleepiness) and MACE (Table 2).

**Table 2:** 10-year incidence of major adverse cardiovascular events (MACE) and all-cause mortality

		MACE, *N* = 3052		All-cause mortality, *N* = 3305	
		*n* = 382		*n* = 510	
		n (%)	p*	n (%)	p*
*Age*					
Middle-aged		48 (3.2)	< 0.01	38 (2.5)	< 0.01
Old		334 (21.3)		472 (26.2)	
*Sex*					
Women		141 (8.9)	< 0.01	180 (10.8)	< 0.01
Men		241 (16.5)		330 (20.2)	
*Body mass index (kg/m*^*2*^*)*					
< 30		340 (12.5)	0.89	443 (15.1)	0.21
≥ 30		42 (12.3)		67 (17.6)	
*Waist-hip ratio* ^a^					
Not obese		135 (8.7)	< 0.01	177 (10.9)	< 0.01
Obese		247 (16.5)		332 (19.8)	
*Smoking history*					
Never		108 (9.5)	< 0.01	113 (9.4)	< 0.01
Ex		169 (15.3)		244 (19.6)	
Current		99 (13.3)		144 (18.3)	
*Hypertension* ^b^					
Normotensive, no BP medication		109 (6.5)	< 0.01	183 (10.4)	< 0.01
Normotensive, on BP medication		18 (16.1)		43 (29.1)	
Hypertensive, on BP medication		79 (24.2)		103 (25.6)	
Hypertensive, no BP medication		176 (18.8)		181 (18.4)	
*Diabetes*					
No		352 (11.9)	< 0.01	454 (14.3)	< 0.01
Yes		30 (30.9)		56 (44.4)	
*Total cholesterol (mmol/l)*					
< 7.0		284 (11.4)	< 0.01	409 (15.1)	0.24
≥ 7.0		98 (17.6)		100 (17.0)	
*HDL cholesterol* ^c^					
Normal		261 (12.0)	0.21	340 (14.7)	0.07
Low		121 (13.7)		169 (17.2)	
*Chronic airway obstruction* ^d^					
FEV_1_/FVC ≥ 0.7		313 (11.2)	< 0.01	384 (12.8)	< 0.01
FEV_1_/FVC < 0.7		69 (27.4)		126 (41.6)	
*Symptoms of sleep apnea*					
Snoring	No	311 (12.3)	0.39	435 (15.9)	0.13
	Yes	71 (13.7)		75 (13.3)	
Breathing cessations	No	377 (12.7)	0.04	497 (15.5)	0.55
	Yes	5 (5.5)		13 (13.3)	
Daytime sleepiness	No	355 (13.0)	0.03	461 (15.6)	0.54
	Yes	27 (8.6)		49 (14.3)	
“OSA cases”^e^	No	368 (12.7)	0.27	491 (15.6)	0.24
	Yes	14 (9.6)		19 (12.1)	

*Chi-Square test BP, blood pressure; HDL, high-density lipoprotein; FVC, forced vital capacity; FEV1, forced expiratory volume in one second; OSA, obstructive sleep apnea

^a^Cut-offs of 0.85 and 0.90 were used to define obesity in women and men, respectively

^b^Hypertension defined by measured systolic blood pressure of ≥140 mmHg and/or diastolic blood pressure of ≥90 mmHg

^c^Low HDL cholesterol was defined by levels below 1.3 mmol/l in women, and by levels below 1.0 mmol/l in men

^d^Spirometry was done 20 minutes after inhalation of 400 μg Salbutamol

^e^”OSA cases” defined by self-reported co-occurrence of snoring, breathing cessations, and daytime sleepiness

In the Cox regression analyses, all the adjustment variables demonstrated significant HRs for both outcomes in crude and multivariate analyses, with the exceptions of obesity (defined by an elevated waist-hip ratio), high total cholesterol, and low HDL cholesterol. Obesity was a significant risk factor for both outcomes only in crude analyses. High total cholesterol was a predictor for MACE in both crude and multivariate analyses but did not show a statistically significant association with all-cause mortality. Low HDL cholesterol was significantly associated with increased risk of both MACE and all-cause mortality, but only in multivariate analyses. CAO was a significant risk factor for both MACE and all-cause mortality in all analyses. There were no positive relationships between any of the OSA variables and MACE, but negative associations were found for daytime sleepiness in crude analysis, and for breathing cessations in multivariate analysis. In the multivariate analysis for all-cause mortality, only daytime sleepiness had a statistically significant HR for the outcome (Table 3).

**Table 3:** Crude and adjusted* hazard ratios for major adverse cardiovascular events and all-cause mortality

	MACE, *N* = 3052				All-cause mortality, *N* = 3305			
	Crude		Adjusted		Crude		Adjusted	
	HR	95% CI	HR	95% CI	HR	95% CI	HR	95% CI
*Age*								
Middle-aged	1		1		1		1	
Old	7.85	5.80–10.62.80.62	6.32	4.52–8.84	11.75	8.45–16.36	12.21	8.52–17.51
*Sex*								
Women	1		1		1		1	
Men	2.01	1.63–2.47	1.85	1.44–2.38	1.97	1.65–2.37	1.64	1.31–2.05
*Waist-hip ratio* ^a^								
Not obese	1		1		1		1	
Obese	2.03	1.65–2.50	1.01	0.79–1.29	1.90	1.59–2.28	0.93	0.75–1.15
*Smoking history*								
Never	1		1		1		1	
Ex	1.72	1.35–2.19	1.24	0.95–1.61	2.20	1.76–2.75	1.50	1.18–1.92
Current	1.49	1.14–1.96	2.09	1.56–2.79	2.06	1.61–2.64	2.80	2.14–3.66
*Hypertension* ^b^								
Normotensive, no BP medication	1		1		1		1	
Normotensive, on BP medication	2.78	1.69–4.58	1.72	1.04–2.85	3.15	2.26–4.40	1.67	1.18–2.37
Hypertensive, on BP medication	4.28	3.20–5.72	1.82	1.33–2.50	2.66	2.09–3.39	1.09	0.84–1.41
Hypertensive, no BP medication	3.19	2.51–4.05	1.67	1.30–2.15	1.85	1.51–2.28	0.99	0.80–1.23
*Diabetes*								
No	1		1		1		1	
Yes	3.26	2.25–4.74	1.83	1.25–2.66	3.83	2.90–5.06	2.34	1.76–3.11
*Total cholesterol (mmol/l)*								
< 7	1		1		1		1	
≥7	1.62	1.28–2.03	1.31	1.03–1.66	1.14	0.92–1.42	0.94	0.75–1.19
*HDL cholesterol* ^c^								
Normal	1		1		1		1	
Low	1.14	0.92–1.41	1.27	1.01–1.59	1.17	0.97–1.40	1.26	1.04–1.54
*Chronic airway obstruction (CAO)* ^d^								
FEV_1_/FVC ≥ 0.7	1		1		1		1	
FEV_1_/FVC < 0.7	3.05	2.35–3.95	1.48	1.12–1.97	3.88	3.17–4.75	1.78	1.44–2.22
*Symptoms of sleep apnea*								
Snoring	1.12	0.86–1.45	1.06	0.82–1.38	0.83	0.65–1.07	0.83	0.65–1.07
Breathing cessations	0.42	0.17–1.01	0.37	0.15–0.90	0.85	0.49–1.48	0.78	0.45–1.35
Daytime sleepiness	0.66	0.45–0.97	0.95	0.63–1.44	0.93	0.69–1.24	1.37	1.01–1.85
“OSA cases”^e^	0.75	0.44–1.28	0.78	0.46–1.34	0.77	0.49–1.22	0.85	0.53–1.34

*Adjusting variables were age, sex, waist-hip ratio, smoking history, hypertension, total cholesterol, HDL cholesterol, and CAO. The symptoms of sleep apnea variables were added separately.

MACE, major adverse cardiovascular event; HR, hazard ratio; CI, confidence interval; BP, blood pressure; HDL, high-density lipoprotein; FEV1, forced expiratory volume in one second; FVC, forced vital capacity; OSA, obstructive sleep apnea

^a^Cut-offs of 0.85 and 0.90 were used to define obesity in women and men, respectively

^b^Hypertension defined by measured systolic blood pressure of ≥140 mmHg and/or diastolic blood pressure of ≥90 mmHg

^c^Low HDL cholesterol was defined by levels below 1.3 mmol/l in women, and by levels below 1.0 mmol/l in men

^d^Spirometry was done 20 minutes after inhalation of 400 μg Salbutamol

^e^"OSA cases" defined by self-reported co-occurrence of snoring, breathing cessations, and daytime sleepiness

**Fig. 2:**
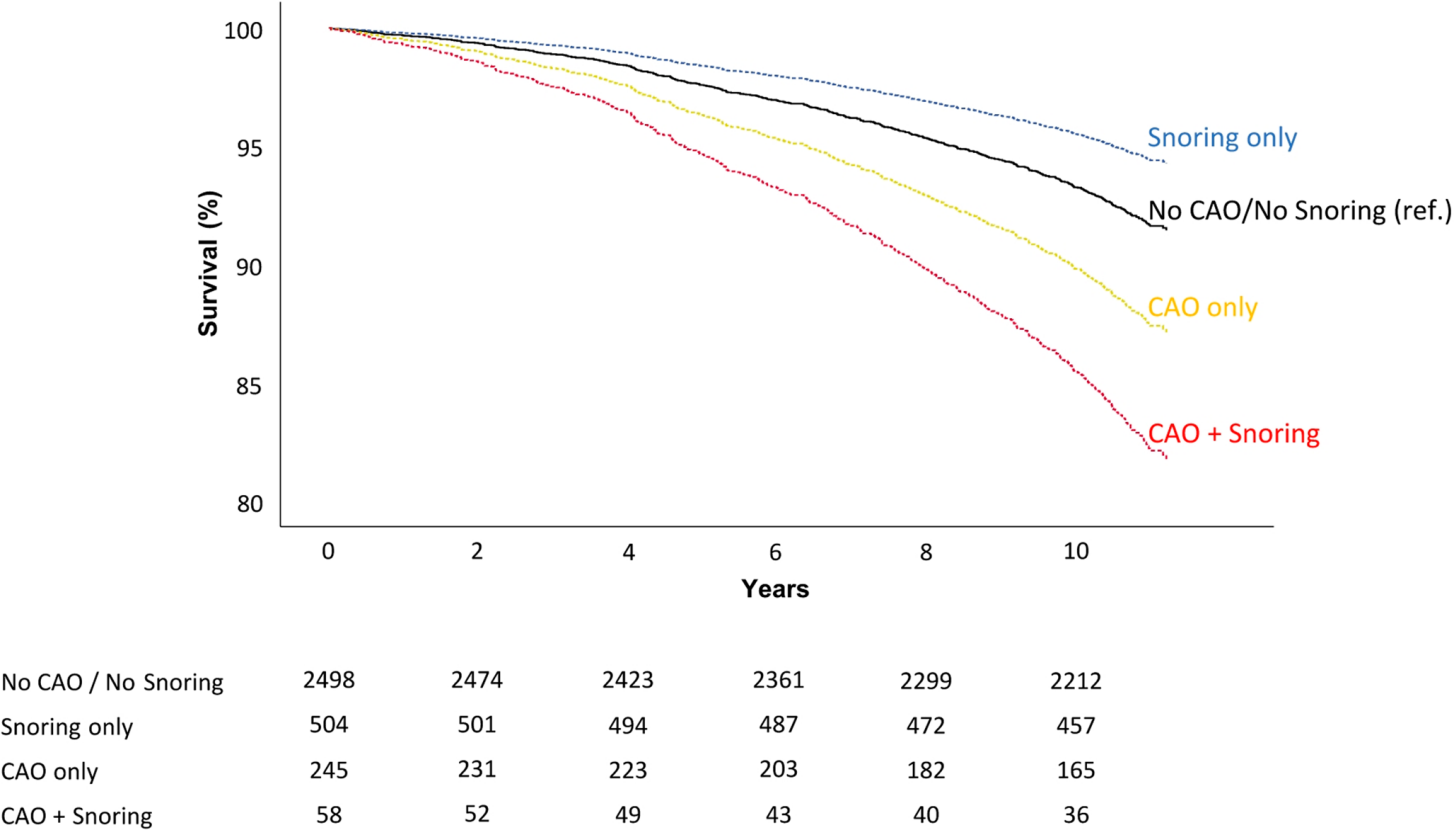
Adjusted Kaplan–Meier survival curves for all-cause mortality stratified by chronic airway obstruction (CAO, post-bronchodilator FEV₁/FVC < 0.70) and habitual snoring. Numbers at risk are shown beneath the x-axis. Compared with the reference group (no CAO, no snoring), adjusted Cox regression hazard ratios (95% CI) were: CAO only 1.54 (1.21–1.96, *p* < 0.001), snoring only 0.66 (0.48–0.90, *p* = 0.009), and CAO + snoring 2.25 (1.52–3.35, *p* < 0.001)

Multivariate interaction analyses revealed no statistically significant interactions between any of the OSA symptoms, CAO, and MACE. For all-cause mortality, there was a statistically significant interaction between snoring and CAO (Table 4). To explore this interaction, a new variable was created by dividing the study population into four groups based on the presence or absence of CAO and snoring. Using this variable, both crude and adjusted analyses revealed a higher risk of all-cause mortality in snorers with CAO, compared to the other groups (Fig. 2). Participants with snoring and no CAO had a significantly lower risk of death, compared to participants with no snoring and no CAO.

**Table 4:** Crude and adjusted* interaction analyses between symptoms of obstructive sleep apnea and chronic airway obstructiona for major adverse cardiovascular events and all-cause mortality

	MACE, *N* = 3052						All-cause mortality, *N* = 3305					
	Crude			Adjusted			Crude			Adjusted		
Interaction term	HR	95% CI	*p*	HR	95% CI	*p*	HR	95% CI	*p*	HR	95% CI	*p*
Snoring × CAO	0.99	0.51–1.93	0.98	1.00	0.51–1.96	1.00	1.89	1.13–3.18	0.02	2.22	1.31–3.76	< 0.01
Breathing cessations × CAO	1.08	0.12–9.80	0.95	0.90	0.10–8.24	0.93	1.33	0.40–4.38	0.64	1.33	0.40–4.42	0.65
Daytime sleepiness × CAO	0.80	0.23–2.72	0.72	0.65	0.19–2.26	0.50	1.00	0.49–2.07	1.00	0.78	0.36–1.67	0.52
“OSA cases”^b^ × CAO	1.45	0.44–4.77	0.54	1.54	0.46–5.13	0.48	1.06	0.39–2.84	0.91	1.03	0.38–2.78	0.96

*Adjusting variables were age, sex, waist-hip ratio, smoking history, hypertension, diabetes, total cholesterol, and HDL cholesterol

MACE, major adverse cardiovascular event; HR, hazard ratio; CI, confidence interval; CAO, chronic airway obstruction; OSA, obstructive sleep apnea

^a^CAO defined by postbronchodilator FEV_1_/FVC<0.7 (spirometry done 20 minutes after inhalation of 400 µg Salbutamol)

^b^"OSA cases" defined by self-reported co-occurrence of snoring, breathing cessations, and daytime sleepiness

A summary of the statistically significant associations between predictors and outcomes is presented in Table 5, complementing the full regression results shown in Tables 3 and 4.

**Table 5:** Significant associations between predictors and outcomes (multivariate Cox regression analyses)

Predictor	Outcome	HR (95% CI)	*p*-value
Chronic airway obstruction (FEV_1_/FVC < 0.70)	MACE	1.48 (1.12–1.97)	0.006
Chronic airway obstruction (FEV_1_/FVC < 0.70)	All-cause mortality	1.78 (1.44–2.22)	< 0.001
Excessive daytime sleepiness	All-cause mortality	1.37 (1.01–1.85)	0.045
Snoring × CAO (interaction)	All-cause mortality	2.22 (1.31–3.76)	0.003

Adjustment variables: age, sex, waist-hip ratio, smoking history, hypertension, diabetes, total cholesterol, and HDL cholesterol

MACE, major adverse cardiovascular event; HR, hazard ratio; CI, confidence interval; CAO, chronic airway obstruction

## Discussion

In this longitudinal study of community-dwelling middle-aged and older adults, CAO significantly increased the risk of both MACE and all-cause mortality. Among OSA symptoms, only EDS independently predicted all-cause mortality. Interaction analysis revealed a combined effect from CAO and habitual snoring on all-cause mortality, suggesting an elevated risk for subjects with obstructive lung disease and coexistent snoring.

Our results align with extensive evidence that COPD increases cardiovascular risk independently of smoking and other risk factors [27–29]. The excess risk is often attributed to systemic inflammation, oxidative stress, vascular dysfunction, and heightened sympathetic activation [10]. Similarly, OSA has been consistently associated with increased cardiovascular morbidity and mortality, particularly in severe cases [30]. The co-occurrence of both conditions, referred to as the overlap syndrome, has been postulated to amplify these mechanisms and accelerate disease progression [10]. Our finding of a synergistic effect between CAO and snoring supports this hypothesis.

Furthermore, consistent with previous studies [31], EDS was independently associated with increased risk of all-cause mortality.

To our knowledge, this is the first general population study to demonstrate an interaction between lung function impairment and a common OSA symptom in predicting long-term survival. This finding appears to contrast with the results reported by Putcha et al., who in their analysis of data from SHHS observed a negative interaction between OSA and reduced lung function [17]. This divergence in findings may be attributed to several factors. Firstly, our study focused on the combined impact of obstructive lung function and common symptoms of OSA, whereas Putcha et al. mainly evaluated the interaction between diagnosed OSA and decreased lung function, measured as decline in FEV_1_. A reduced FEV_1_ does not solely signify obstructive lung disease; it may also stem from a range of conditions linked to a restrictive lung function, such as heart failure, interstitial lung disease, obesity, or neuromuscular diseases. In the SHHS models, the FEV_1_/FVC ratio, not using post-bronchodilator values, exhibited a much weaker interaction than FEV_1_ with the AHI and all-cause mortality, with most estimates being nonsignificant. These results were described as “likely caused by the narrow distribution of the ratio and by a limited number of participants with low FEV_1_/FVC values” [17]. Secondly, in our study, enriched by an older cohort, and using post-bronchodilator values, a high proportion of participants had CAO, making more robust analyses of COPD and OSA symptoms possible. As acknowledged by the authors [17], the SHHS cohort was not enriched with participants with significant lung disease and thus could not provide evidence on health-related outcomes, such as all-cause mortality, in individuals with OSA and COPD. Finally, the variance in outcomes could be reflective of the different clinical implications of snoring in the absence of diagnosed OSA, compared to the presence of sleep apnea itself. Nevertheless, our findings suggest that the coexistence of obstructive lung disease and snoring might represent a distinct clinical phenotype with a unique risk profile.

Contrary to several previous studies that have reported OSA to be associated with elevated cardiovascular risk [30], we found no significant associations between self-reported OSA symptoms and MACE. Furthermore, there were no positive interactions between CAO and any of the OSA symptoms in relation to this endpoint. By comparison, Kendzerska et al. (2019) reported in a large clinical cohort that COPD combined with nocturnal hypoxemia was associated with increased cardiovascular and mortality risk [16]. However, when OSA was defined by the apnea-hypopnea index, the coexistence of COPD and severe OSA was associated with a lower cardiovascular risk than COPD alone [16]. We believe several factors need to be taken into consideration when explaining why our results differ from previous studies. Firstly, we applied a classical 3-point MACE definition (nonfatal myocardial infarction, nonfatal stroke, and cardiovascular death). Other studies have used broader composites including heart failure, revascularization procedures, and atrial fibrillation [32]. While such definitions increase event numbers, they may dilute specificity by combining heterogeneous outcomes. We chose the narrower definition to ensure greater clinical specificity, though this reduced the number of events and may have limited statistical power. Secondly, as observed in a previous study, although highly prevalent, most individuals suffering from severe OSA are neither symptomatic nor sleepy [33]. Therefore, we cannot compare our results to those of studies applying polygraphy or polysomnography for an OSA diagnosis. On the other hand, our findings are, in part, supported by results from the European Sleep Apnoea Database, which have revealed a lower prevalence of cardiovascular disease among sleepy OSA patients, compared to other OSA phenotypes [34].

Interestingly, we found that snoring alone, without concurrent CAO, was associated with lower risk of all-cause mortality. This counterintuitive result may reflect survivorship bias, particularly in the older cohort, as vulnerable individuals could have died before baseline, leaving a subset of resilient “survivor snorers”. This may represent a phenotype of habitual snoring without OSA, which could explain the apparent protective association.

This study’s strengths include a high participation rate (69%), single-operator post-bronchodilator spirometry performed on all participants, and reliance on registry-based outcomes. A limitation of the study was the reliance on self-reported OSA symptoms, as polysomnography was not feasible in this large cohort. This approach, however, is consistent with other population-based studies and captures symptom burden, which may itself be independently linked to adverse cardiovascular outcomes [35]. We do, however, acknowledge the limitations of this approach and highlight the need for future studies employing objective sleep assessments. Another limitation was the lack of data on OSA diagnosis and treatment, leaving us unable to determine the number of study participants that received CPAP or other treatments. However, we believe this number was likely low, as OSA was largely underdiagnosed in Norway in the late 1990s, and CPAP was not commonly prescribed.

In conclusion, our findings confirm that CAO and EDS are independent predictors of all-cause mortality in community-dwelling adults. More importantly, we demonstrate a synergistic association between CAO and habitual snoring, suggesting that coexistence of obstructive lung disease and OSA symptoms identifies a particularly vulnerable subgroup. These results underscore the importance of recognizing OSA-related symptoms in patients with COPD and provide a rationale for further studies using objective sleep assessments to clarify mechanisms, refine risk prediction, and guide preventive strategies.

## Data Availability

The data that support the findings of this study are not openly available due to reasons of sensitivity. Data are in controlled access data storage at the University of Bergen and are available from the corresponding author upon reasonable request.
